# Challenges in Diagnosis and Treatment of Lung Cancer in People with Intellectual Disabilities: Current State of Knowledge

**DOI:** 10.1155/2016/6787648

**Published:** 2016-09-26

**Authors:** Daniel Satgé, Emmanuelle Kempf, Jean-Bernard Dubois, Motoi Nishi, Jean Trédaniel

**Affiliations:** ^1^Oncodéfi, Parc Euromédecine, 209 avenue des Apothicaires, Parc Euromédecine, 34 090 Montpellier, France; ^2^Team Biostatistics Epidemiology Public Health, EA 2415, University Institute for Clinical Research, 34 093 Montpellier, France; ^3^Medical Oncology Department, Hôpital Henri Mondor, AP-HP, 51 avenue du Maréchal de Lattre de Tassigny, 94 010 Créteil, France; ^4^Pierre et Marie Curie University, Paris, France; ^5^Radiotherapy Service, Institut de Cancérologie Val d'Aurelle, Parc Euromédecine, 208 avenue des Apothicaires, 34 298 Montpellier, France; ^6^Department of Fundamental Health Sciences, Health Sciences University of Hokkaido, Tobetsu, Hokkaido 061-0293, Japan; ^7^Descartes University, Paris, France; ^8^Unit of Thoracic Oncology Service, Groupe Hospitalier Paris Saint Joseph, 185 rue Raymond Losserand, 75 014 Paris, France

## Abstract

As the life expectancy of people with intellectual disability (ID) has progressed, they have become similarly at risk of cancer as individuals of the general population. Epidemiological studies indicate a reduced incidence and mortality from lung cancer in the total population of persons with ID. However, the pattern is heterogeneous and the risk is strongly correlated with the impairment level; persons with mild intellectual impairment have higher cancer risk, and this subgroup also has the highest tobacco consumption (the major risk factor for lung cancer) compared to individuals with more severe impairment. Clinical presentation of lung cancer in persons with ID is often atypical, with symptoms frequently hidden by the mental state and communication impairments. Treatment can be impeded by incomplete understanding and lack of cooperation on the part of the patient; nevertheless, general principles for treating lung cancer must be applied to persons with ID. Early diagnosis and implementation of an adapted treatment plan may result in lung cancer outcomes similar to those of individuals in the general population. Physicians facing the difficult task of treating lung cancer in persons with ID are called to carry out their mission of care in a responsible, free, and creative way.

## 1. Introduction

As the life expectancy of people with intellectual disability (persons with ID) has increased over the last few decades [1], they have become more exposed to cancer risk [2]. Indeed, evidence suggests that prevalence rates for cancer are increasing among these individuals. Intellectual disability is defined by significant limitations in both intellectual functioning and adaptive behavior, which comprises many everyday social and practical skills, and originates before the age of 18. This definition excludes pure psychiatric conditions such as schizophrenia and mental impairment in late adulthood such as Alzheimer's disease. Thus, persons with ID account for 2% of the general population [3] and represent an important subgroup in which to understand cancer incidence and features.

Persons with ID are reported to have a nearly similar risk of developing cancer as individuals in the general population [4, 5]. However, tumors among this subpopulation are poorly known and differ in frequency from those in the general population [1, 4, 5]. Additionally, the care of persons with ID with cancer presents a challenge to caregivers and clinicians at all stages, from prevention to screening, detection, diagnosis, treatment, and follow-up. In particular, persons with ID have difficulties in understanding the illness and treatment. Care providers are therefore confronted with obstacles in communication, assessment, and patient fear. However, a better familiarity with this population, improved knowledge of how to communicate with affected individuals, and a clearer grasp of their understanding of disease, when combined with a skilled approach to obtaining their cooperation with treatment, will help overcome such challenges. Tools to explain prevention, aid early detection of tumors, and evaluate pain for those with whom communication is difficult are available and are valuable in caring for persons with ID.

The present article focuses on the frequency of lung cancer in persons with ID, its diagnosis, and potential treatment difficulties, seeking to help improve overall clinical care of this important population.

## 2. Methods

A search of the literature was conducted in two phases. First, a regular follow-up search of cancers in persons with intellectual disabilities was conducted for ten years by one author (Daniel Satgé). This search was performed every month on Medline using the words “mental retardation” and “intellectual disability” indexed with “cancer”, “malignancy”, and “tumor”. Second, a more specific search was performed to focus on lung cancer. In PubMed, we searched the terms “mental retardation”, “intellectual disability”, and “learning disability” with each of the terms “lung cancer”, “lung malignancy”, “lung tumor”, “lung carcinoma”, “lung adenocarcinoma”, “lung small cell carcinoma,” “lung carcinoid”, “lung sarcoma”, “lung fibrosarcoma”, “lung lymphoma”, “bronchial carcinoma”, “bronchial adenocarcinoma”, and “pleural mesothelioma”. On Google Scholar we searched the terms “mental retardation”, “intellectual disability”, and “learning disability” with “lung cancer”, “lung malignancy”, and “lung carcinoma”. These searches were not limited in the time of publication or language. Retrieved abstracts were scanned manually, and potentially suitable papers were collected. Cases where cognitive impairment occurred after the age of 18 were excluded, as well as patients with pure psychiatric conditions without intellectual disability. The extracted data were frequency clinical characteristics, treatment, and environmental and genetic risk factors.

## 3. Results and Discussion

### 3.1. Results

The search on PubMed provided 144 abstracts, of which 33 were retained. The search on Google Scholar found 23 abstracts, of which 11 were retained. Among these 44 abstracts selected in the second component of the search, 15 were not found in the first general follow-up search. Additional articles were identified through citations and references. In total, 76 articles were included.

### 3.2. Frequency

The frequency of lung cancer in persons with ID has not been evaluated through targeted studies. Nonetheless, data provided in studies based on all cancers in persons with ID provide helpful indications. The two available epidemiological studies on cancer incidence indicate a reduced frequency of lung cancer in the total population of persons with ID [4, 5]. A report from Finland observed 17 cases; 27.6 cases were expected; thus, the standardized incidence ratio (SIR) was significantly lower at 0.6 (95%, CI = 0.4–1.0) [4]. In Australia a statistically nonsignificant decrease in lung cancer incidence was reported, with an SIR of 0.63 for men (5 cases observed for 7.90 expected, 95% CI = 0.21–1.48) and 0.44 for women (2 cases observed for 4.56 expected, 95%, CI = 0.05–1.58) [5].

Mortality data also indicate a lower frequency of lung cancer in persons with ID. A recent evaluation conducted in Leicester County, United Kingdom (UK), revealed that persons with moderate, severe, and profound ID exhibit a standardized mortality ratio (SMR) for all cancers similar to that in the general population, at 0.94. However, the SMR for lung cancer is reduced to 0.62 (6 cases observed for 9.6 expected, 95%, CI = 0.22–1.36) [6]. In the Stoke Park Hospital in Bristol, UK, three consecutive studies on cancer death indicated an underrepresentation of lung-bronchus cancer compared to the general population. These pulmonary malignancies accounted for just 3/81 deaths in the period 1936–1971, 2/53 deaths in the period 1976–1985, and 2/27 deaths for the period 1986–1995 (cumulative: 4.3% of all cancer deaths) [7–9]. Similarly, among 19 cancer deaths in an institution in the rural southeastern United States (US) during the years 1974–1985, none were related to the lung [10]. In Israel, 8 of 74 registered cancer deaths (10.8%) in persons with ID living in residential care centers and in the community during the years 1991–2005 were attributed to lung cancer [11]. Finally, an evaluation of morbidity in elderly people living in the Hooge Burch Centre in the Netherlands reported 17 cancers among 77 residents aged 60 years and over, which was nearly the same as expected in the general population for all cancers. Two (12%) of these were lung carcinoma in men [12], indicating a lower frequency of lung cancer compared with the general population.

Further, the risk for lung cancer in persons with ID strongly correlates with the severity of ID. In the Australian study, 6 of 7 lung cancers occurred in individuals with mild ID [5]. In the Finish study, no lung cancer was observed in individuals with severe and profound ID, while 5.2 were expected, and 17 lung cancers were noted compared to 20.4 expected in individuals with moderate to mild ID (SIR = 0.8, 95%, CI = 0.4–1.2) [4]. Similarly, in a series of 79 cancer deaths in persons with moderate, severe, and profound ID collected in 8 Californian institutions during the years 1979–1986, only one was attributed to lung cancer [13], although lung cancer was, during the same period, a leading cause of cancer death in the general population.

Globally, the risk for lung cancer appears, in these studies, to be lower in persons with ID compared to the general population. We do not exclude the possibility of an underestimation of the burden of lung cancer, particularly in older studies. This is possible because, first, persons with ID had a shorter life expectancy only a few decades ago. Similarly, diagnostic tools, particularly medical imaging, were less available and effective during that time. However, digestive tract cancers were frequent in the same studies, indicating that other types of malignancies were not missed. Thus, we believe that significant underevaluation of the frequency of lung cancer is unlikely. Additionally, these works included a high proportion of individuals with moderate and severe ID in whom lung cancer is rare, which may lead to the false assumption that lung cancer is very rare in all persons with ID, including persons with mild ID. On the basis of the current literature, we may say that lung cancer appears less frequent in persons with ID compared to the general population, particularly for the 20% of those with profound, severe, and moderate ID and to a lesser extent for those with mild ID. Targeted studies are needed to assess the real risk of lung cancer in persons with ID. Various histological types have been reported: adenocarcinoma [14], small cell carcinoma [15], carcinoid [16], synovial sarcoma [17], and lymphoma of the lung [18].

### 3.3. Environmental Risk Factors

The major risk factor for lung cancer is tobacco smoking; infrequently, persons with mild ID are exposed to occupational carcinogens such as asbestos and may develop a lung carcinoma [19]. Overall, the prevalence of smoking in the total population of persons with ID is lower than in the general population [20]. Smoking among persons with ID has been reported at high rates in the US (New Jersey) and Australia, with rates of 37% and 36%, respectively; the rate of smoking among the general population is 26% for each of these two countries [21, 22]. Lower rates have been reported in other studies: 2.6% in Ireland [23], 6.2% in UK (Midlands) [24], 7% in the US (Florida) [25], and 9% in Finland [4].

Smoking behavior is also related to the severity of ID; smokers are more common among individuals with mild ID. Further, smoking is more common among group of home residents than among those who live in an institution or those who live in their families, with rates varying from 3.8% to 20.8%. With the closure of long-stay institutions, more persons with ID are living in the community. Drinking alcohol, being sexually active, and having schizophrenia are also significant predictors of smoking among persons with ID [21].

Interventions such as a smoking education course to lower tobacco use have been implemented among persons with mild ID leading to some success [22]. However, research is needed to test the effectiveness of dedicated interventions in lowering tobacco use for this group [20, 26].

### 3.4. Genetic Risk Factors and Protective Factors

The population of persons with ID is highly heterogeneous when considering the cause of ID. In many patients, particularly those with mild ID, the causes remain unknown. For about one-third of patients, ID is attributable to one of nearly 2000 known genetic conditions. Nongenetic causes are perinatal, vascular, postinfectious, toxic, posttraumatic, and environmental [1].

Some studies have assessed cancer incidence among individuals with specific genetic conditions. An excess of lung cancer has been reported among individuals with Klinefelter syndrome in two epidemiological studies [27, 28]. In one study, the statistically significant SMR of 1.5 (95% CI = 1.0–2.0) included an unusually large proportion of small cell tumors. Since men with Klinefelter syndrome have a slightly higher risk of ID, the cooccurrence of lung cancer with ID is sometimes observed [19]. In contrast, three epidemiological studies on cancer incidence among individuals with Down syndrome identified only one lung cancer among 192 malignancies [29–31]. Further, two mortality studies reported an absence of lung cancer in this population, in which 17.68 and 4.39 cases would have been expected, thereby indicating that the condition may protect against lung cancer [32, 33]. Because individuals with Down syndrome carry an extra copy of chromosome 21 (in part or whole), one or more of the 300 genes mapping to this chromosome could be responsible for the hypothesized protective effect [34]. Similarly, in fragile-X syndrome, the most common form of inherited ID, only two lung tumors have been reported [35–37]. This condition seems to protect against cancer in many organs, generating the hypothesis of a protective effect of the* FMRP* gene [38]. Finally, women with Turner syndrome, who have a slightly higher risk for ID, have been estimated to exhibit a statistically nonsignificant lower risk of lung cancer (SIR = 0.7, 95% CI = 0.2–1.8) [39]. The reduced frequency of lung cancer observed in individuals with these genetic conditions may, in part, explain the lower incidence of lung tumors observed in the entire population of persons with ID.

Children and young adults with other genetic conditions, such as Nijmegen syndrome [18] and ataxia telangiectasia [40], that increase the risk for lymphoma have been reported to present with lung lymphoma. Additionally, persons with tuberous sclerosis frequently develop pulmonary lymphangioleiomyomatosis [41].

### 3.5. Clinical Presentation

Little data are available to document the clinical presentation of lung cancer in persons with ID. Presentation may be similar to that of nondisabled persons: cough, dyspnea, hemoptysis, and chest pain. However, symptoms are frequently concealed by the mental state and communication impairments. In particular, because many people with ID communicate pain and unease in unconventional ways that may not be readily understood or may not appear to express pain at all, a tumor may remain undiagnosed for some time. Among eight available reports with clinical history, two bronchial carcinomas were discovered on routine chest radiographs before a surgical procedure for another disease [14, 16]. One of these patients complained of minor chest pain despite bilateral spread of the tumor, but his family did not believe it was a serious problem. The treatment team was surprised that he did not develop pulmonary and cardiovascular symptoms [14]. For two patients, the tumor was found in the context of recurrent infections [42, 43]. The fifth tumor was revealed by an acute pneumothorax [17]. In a sixth patient with a metastatic tumor, the cancer was diagnosed in a context of low back pain for four months and unexplained weight loss [44]. The seventh patient, a 47-year-old man with mild ID who smoked tobacco, presented with shortness of breath and cough. Because he had a history of imagining illnesses, his family and doctors did not believe him when he first complained of chest pain [45, 46]. The eighth patient, a 22-year-old woman with Sotos syndrome, was admitted at a metastatic stage one month before death with significant weight loss, cough, dyspnea, and lethargy [15].

In general, the more severe the disability, the less clear the symptomatology, thereby introducing diagnostic challenges for patients with severe and profound ID. A patient who is unable to express his symptoms may present with mood and behavior modifications. He may behave aggressively, as was described in a 41-year-old man with a brain tumor [47], or remain unusually uncommunicative. These presentations incorrectly suggest a mental health issue. Further, some patients may be fearful of physical examination and may therefore mask their disease as long as possible [48].

Additionally, families do not have specific knowledge of cancer risk in persons with ID. In residential homes, nursing homes, and supported living arrangements, staff members are less knowledgeable about signs and symptoms of cancer than is optimal [45, 49]. The difficulty in making a diagnosis and the lack of knowledge of families and professional caregivers may introduce diagnosis delays and underdiagnoses. For example, two lung cancers were found in individuals at an institution: one too late for treatment and the other at autopsy [12].

Indeed, diagnosis delay is not specific to lung cancer and has been observed in a series of breast carcinomas [50] and in a review of oral malignancies [51]. A way to overcome diagnosis difficulties is to remain closely attentive to the patient presentation and complaints [44, 52] and, above all, fully consider caregiver and family reports on the patient [53].

### 3.6. Treatment

General principles for treating lung cancer must be applied to persons with ID. Treating a lung tumor is more difficult in adults with ID as compared with nondisabled persons. This is because, first, for many patients with ID the cancer diagnosis is delayed. When tumors are discovered at a late stage they must be treated with more intense therapeutic regimens [12, 45, 54]. Second, communication barriers and cognitive awareness introduce difficulties in providing the patient with an understanding of the disease and obtaining the patient's cooperation in the treatment process [55]. In this regard, radiotherapy may be discarded if a patient cannot remain still or is particularly vulnerable to radiation. Third, biochemical difficulties linked to some genetic conditions can limit the use of certain drugs [1]. Chemotherapy may also need to be modified or adapted because of vulnerability to certain chemical agents. Additionally, surgical intervention is riskier in cognitively impaired patients, who have a higher likelihood of developing postoperative complications [54–56]. Administering anesthesia is also more complicated and must be modified for some underlying genetic conditions. A recent study showed that persons with mental health disorders or ID and newly diagnosed with stage I non-small cell lung cancer had significantly lower surgical rates as compared with nondisabled persons [57]. They also had a higher use of radiotherapy, described by the authors as reserved for persons with medical contraindications to surgical treatment. Indeed, case reports illustrate the situation: chemotherapy was discarded in two patients due to their mental state [14, 58], and two others received half doses of chemotherapy due to ataxia telangiectasia-like disorder [59]. In a fifth patient, ifosfamide was discarded since he had a history of seizures [17]. Nonetheless, two patients were successfully treated using bilobectomy for a stage I and a stage IIB carcinoid tumor, illustrating the potential to cure a patient with ID [16, 43]. The provision of palliative care to persons with ID is a fundamental point but is beyond the scope of this article.

### 3.7. Ethical Issues

Communication between cancer patients and their caregivers is likely to be poor with persons with ID [55]. In a study of 13 persons with ID with cancer, the diagnosis was disclosed to 11 of them but only 2 fully understood it; caregivers seemed to have done little to help foster the understanding of cancer diagnosis and prognosis implications [60]. Different strategies have been identified to improve communication between hospitalized persons with ID and caregivers [61], and some are dedicated to cancer [62]. Persons with ID may have an inequitable access to cancer prevention, screening, treatment, and clinical research [49, 63–65]. Further, a protectionist interpretation of the Hippocratic principle of nonmalfeasance may lead to an undertreatment of persons with ID with cancer [66]. Taking care of persons with ID with cancer imposes questions on caregivers' habits and increases doubts and uncertainties; however, specific training seems to improve caregivers' confidence and comfort when facing such patients with cancer [67].

### 3.8. Study Limitation and Future Studies

The review examines an important and underrecognized topic that deserves our attention. As the life expectancy of persons with ID has significantly progressed, these people are now at risk of various cancers. We acknowledge that this first review on the subject has a number of limitations linked to the available literature. Although a 10-year follow-up of the literature on cancer in persons with ID in PubMed and in books completed by a systematic search with targeted keywords has been conducted, a limited number of articles were collected. Epidemiological data on cancer incidence and on cancer mortality are not focused on lung cancer. Such a focused study could provide additional data on cancer occurrence according to ID level, to the place of residence, and to associated risk factors. Studies in institutions that were conducted decades ago may result in an underevaluation of cancers, including lung cancers. However, as other cancers such as digestive tract malignancies have been reported in these works, it is likely that the underestimation of lung cancer is not significant. On the other hand, few case reports presenting unusual tumors in rare syndromes may lead to an overestimation of unusual histological types of lung cancer. Further, the few detailed reports do not enable us to draw conclusions on symptoms that lead to diagnosis or on treatment results. These gaps in knowledge show the need for clinical series that document symptoms, tumor histology, treatment compliance, and treatment results.

## 4. Conclusion

Lung cancer appears to be less frequent in persons with ID compared to the general population, based on available literature. Lung cancer seems even rarer in persons with moderate, severe, and profound ID. As lung cancer is relatively uncommon in persons with ID and since symptoms are often atypical and less noticeable, diagnoses are often delayed. The possibility of a lung malignancy should be considered, particularly for persons with ID who smoke tobacco. Further efforts should be undertaken to lower their tobacco use. Treatment is difficult due to psychological and sometimes biological obstacles, which require adaptations to standard care plans. Two examples of carcinoid tumors show that lung cancer treatment outcomes can be as good among persons with ID as in the general population when the tumor is discovered early, when adapted care is given and if the person is able to cope with treatment.
